# Abnormal regional activity in the prefrontal‐limbic circuit at rest: Potential imaging markers and treatment predictors in drug‐naive anxiety disorders

**DOI:** 10.1111/cns.14523

**Published:** 2023-11-21

**Authors:** Xiaoxiao Shan, Haohao Yan, Huabing Li, Feng Liu, Ping Li, Jingping Zhao, Wenbin Guo

**Affiliations:** ^1^ Department of Psychiatry, National Clinical Research Center for Mental Disorders, and National Center for Mental Disorders The Second Xiangya Hospital of Central South University Changsha Hunan China; ^2^ Department of Psychiatry, Shandong Mental Health Center Shandong University Jinan Shandong China; ^3^ Department of Radiology The Second Xiangya Hospital of Central South University Changsha Hunan China; ^4^ Department of Radiology Tianjin Medical University General Hospital Tianjin China; ^5^ Department of Psychiatry Qiqihar Medical University Qiqihar Heilongjiang China

**Keywords:** fractional amplitude of low‐frequency fluctuation, generalized anxiety disorder, magnetic resonance imaging, panic disorder, support vector regression

## Abstract

**Background:**

Previous research has identified functional impairments within the prefrontal‐limbic circuit in individuals with anxiety disorders. However, the link between these deficiencies, clinical symptoms, and responses to antipsychotic treatment is still not fully understood. This study aimed to investigate abnormal regional activity within the prefrontal‐limbic circuit among drug‐naive individuals diagnosed with generalized anxiety disorder (GAD) and panic disorder (PD) and to analyze changes following treatment.

**Methods:**

Resting‐state magnetic resonance imaging was performed on a cohort of 118 anxiety disorder patients (64 GAD, 54 PD) and 61 healthy controls (HCs) at baseline. Among them, 52 patients with GAD and 44 patients with PD underwent a 4‐week treatment regimen of paroxetine. Fractional amplitude of low‐frequency fluctuation (fALFF) measurements and pattern classification techniques were employed to analyze the data in accordance with the human Brainnetome atlas.

**Results:**

Both patients with GAD and PD demonstrated decreased fALFF in the right cHipp subregion of the hippocampus and increased fALFF in specified subregions of the cingulate and orbitofrontal lobe. Notably, patients with PD exhibited significantly higher fALFF in the left A24cd subregion compared to patients with GAD, while other ROI subregions showed no significant variations between the two patient groups. Whole‐brain analysis revealed abnormal fALFF in both patient groups, primarily in specific areas of the cingulate and parasingulate gyrus, as well as the inferior and medial orbitofrontal gyrus (OFG). Following a 4‐week treatment period, specific subregions in the GAD and PD groups showed a significant decrease in fALFF. Further analysis using support vector regression indicated that fALFF measurements in the right A13 and right A24cd subregions may be predictive of treatment response among anxiety disorder patients.

**Conclusions:**

Aberrant functional activity in certain subregions of the prefrontal‐limbic circuit appears to be linked to the manifestation of anxiety disorders. These findings suggest potential imaging indicators for individual responses to antipsychotic treatment.

## Graphical abstract

This study aims to explore the regional activity within the prefrontal‐limbic circuit among drug‐naive individuals diagnosed with GAD and panic disorder (PD) and to analyze changes following treatment. To our knowledge, this study is the first to explore the regional activity within the prefrontal‐limbic system circuitry using a finely tuned Brainnetome atlas. A major strength of this study is that the abnormal regional activity in the right cHipp subregion of the hippocampus, left A23d and bilateral A32sg subregions of the cingulate, as well as the left A14m and bilateral A12/47L subregions of the orbitofrontal lobe, may be related to anxiety‐induced state changes. In addition, high fALFF levels in the right A13 and right A24cd subregions could serve as predictors for individual responses to antipsychotic therapy.

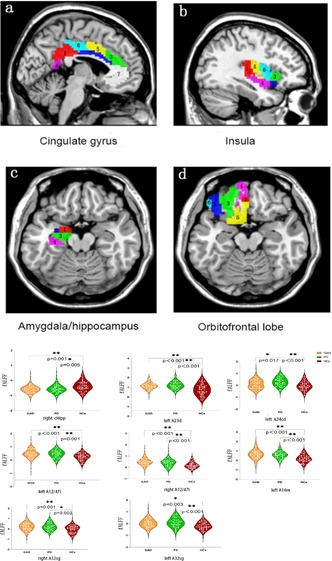

## 1 INTRODUCTION

Anxiety disorders, characterized by fear, worry, and tension, are among the most prevalent mental health conditions globally, often presenting with concomitant autonomic nervous symptoms such as palpitations and chest tightness. Existing systematic reviews and meta‐analyses establish a global prevalence of anxiety disorders at 7.3% (ranging between 4.8% and 10.9%),^1^, ^2^ underlining their significance as a public health concern. Despite numerous neuroimaging studies investigating the pathological foundations of anxiety disorders, the core mechanisms underlying these conditions remain elusive.

In the context of neural connectivity, mounting evidence suggests that the neural pathways linking the prefrontal cortex (PFC) to limbic regions—including the amygdala, anterior cingulate gyrus (ACC), hippocampus, and insula—are disrupted in individuals with anxiety disorders. Task‐state fMRI studies indicate that patients with generalized anxiety disorder (GAD) demonstrate enhanced responses to fear‐inducing faces during facial emotional tasks. These activations are detected within the prefrontal‐limbic network, encompassing the amygdala, ACC, and PFC. Functional connectivity (FC) analysis reveals a positive correlation among these regions.^3^ Increased activation in the amygdala, thalamus, insula, ACC, and midcingulate cortex have been found in patients with PD when presented with terror stimuli.^4^ A recent meta‐analysis revealed alterations in neural activity and FC across multiple brain regions of the prefrontal‐limbic circuit, such as the amygdala, dorsal prefrontal cortex, ACC, and hippocampus, in individuals with GAD.^5^ Resting‐state study focusing on connectivity between the amygdala and the DMN and salient network, found increased FC in critical regions, including the amygdala and cingulate cortex, and decreased FC in the frontal and temporal cortex.^6^ It is worth noting that most previous studies on brain function in anxiety disorders have primarily focused on task‐state studies and have been cross‐sectional, with resting‐state and longitudinal studies being relatively sparse. Furthermore, longitudinal studies examining the changes in brain function associated with pharmacological treatment in anxiety disorders remain scarce.

The fractional amplitude of low‐frequency fluctuation (fALFF) is a method employed to discern regional information, which is accomplished by quantifying the ratio of the power spectrum within the low‐frequency range relative to the entire frequency range. It enables the detection of regional intensity of spontaneous BOLD signal fluctuations.^7^ This intensity correlates with spontaneous alterations in regional blood flow, signifying that increases in fALFF might indicate hyperactivity within brain regions, while decreases could signal neural hypoactivity.^8^, ^9^ Enhanced based on the original ALFF method, fALFF is designed to better contend with physiological noise, thereby efficiently mitigating the impact of non‐specific signal elements such as cerebrospinal fluid, respiration, and cardiac rhythm.^7^ This refinement heightens sensitivity and specificity for detecting spontaneous brain activity in target regions. The fALFF technique has been widely used in studies investigating brain function in a variety of psychiatric disorders, including schizophrenia, bipolar disorder, and major depressive disorder (MDD).^8^, ^10^, ^11^

Pattern recognition techniques, such as support vector regression (SVR) and support vector machine, can be utilized for predicting and classifying psychiatric disorders based on neuroanatomical biomarkers. SVR is predominantly employed to address regression and prediction challenges. It offers robust generalization performance and high‐prediction accuracy. These pattern recognition techniques have been applied to distinguish patients with MDD, bipolar disorder, and schizophrenia from HCs and to forecast treatment responses in MDD and schizophrenia.^12^, ^13^

In this investigation, we recruited 64 drug‐naive patients with GAD and 54 drug‐naive patients with PD to examine the anomalies in fALFF within the prefrontal‐limbic system and its post‐treatment modifications. Functional MRI scans and the clinical statuses of patients were gathered at two instances, namely, at baseline and following 4 weeks of treatment. Our initial analysis, grounded in the human Brainnetome atlas, involved a comparison of fALFF across the three groups using regions of interest (ROI) that include the cingulate gyrus, amygdala, insula, hippocampi, and orbitofrontal lobe. This was further substantiated by a whole‐brain analysis to confirm the results. Our hypothesis posited that compared to HCs, patients with GAD and PD would demonstrate aberrant fALFF in the prefrontal‐limbic circuit, potentially correlating with clinical symptomatology. Specifically, drawing upon existing evidence implicating the limbic system in emotional processing and fear responses, we hypothesized that patients with GAD and PD would exhibit elevated fALFF in key regions such as the amygdala, cingulate gyrus, hippocampus, and insula when compared to HCs. Moreover, in light of research indicating compromised top‐down regulatory mechanisms in the prefrontal cortex, we postulated that fALFF would be diminished in the orbitofrontal cortex of both patients with GAD and PD relative to HCs. At last, we hypothesized that if pharmacotherapy exerts modulatory effects on the functional activities of these neural substrates, post‐treatment fALFF metrics within the limbic system would decrease in patients with GAD and PD, while those in the orbitofrontal cortex would show an increase. In addition, we proposed that such alterations in post‐treatment fALFF could serve as predictive markers of therapeutic efficacy, as evaluated through SVR analyses.

## 2 MATERIALS AND METHODS

### 2.1 Participants

A total of 118 drug‐naive patients with anxiety disorders (64 patients with GAD and 54 patients with PD) and 61 healthy controls (HCs) were recruited. in the study. Of these, 52 patients with GAD and 44 patients with PD underwent a 4‐week treatment regimen with paroxetine. The recruitment occurred at the Second Xiangya Hospital of Central South University in China. The diagnoses of anxiety disorders were confirmed by two psychiatrists using the *Diagnostic and Statistical Manual of Mental Disorders, Fifth Edition* (DSM‐5). The inclusion criteria were as follows: (1) Hamilton Depression Scale (HAMD‐17) total scores ≤17, the first depression item score ≤2, and the third suicide item score <2; (2) no previous exposure to psychological or psychotropic medications treatment; and (3) absence of active non‐anxiety psychiatric disorders as per DSM‐5, with no history of other mental disorders. Patients presenting with comorbidity of other anxiety disorder subtypes per diagnostic criteria were excluded. Clinical symptoms were assessed using the Hamilton Anxiety Scale‐14 (HAMA‐14), HAMD‐17, Social Disability Screening Schedule (SDSS), and Simplified Coping Style Questionnaire (SCSQ). The Brief Cognitive Assessment for Schizophrenia (B‐CATS), which incorporates three neuropsychological tests (Digit Symbol Test, Trail Making Test (TMT) parts A and B, and Animal Naming Fluency Test), evaluated cognitive function, including attention, executive speed, language fluency, learning, and memory. All the patients were prescribed paroxetine, with the dosage progressively increased based on the patient's condition within the first week and maintained stable until the final MRI scanning. The use of other antidepressants and benzodiazepines was prohibited.

Sixty‐one HCs, unconnected to the patients and matching them in gender, age, and years of education, were sourced from the local community. HCs were excluded if they exhibited any symptoms of psychosis, substance abuse, or neurological disease. Any potential HCs with a first‐degree relative diagnosed with a psychiatric disorder were also excluded.

The general exclusion criteria for all participants included the following: (1) presence of other mental disorders as per DSM‐5; (2) historical or current serious physical illness, such as cardiovascular, liver, or kidney diseases; (3) evident suicidal tendencies or attempts; (4) any history of traumatic brain injury or epilepsy; (5) severe sleep disorders necessitating sleep medication; (6) contraindications for MRI scan; and (7) pregnancy.

All the participants provided written informed consent after a thorough briefing about the study. The Second Xiangya Hospital of Central South University's ethics committee approved the study, conducted in compliance with the Helsinki Declaration.

### 2.2 Image acquisition and data preprocessing

Image acquisition was facilitated by a Philips Achieva 3.0 T scanner (Philips Medical Systems, Best, Netherlands) at the Second Xiangya Hospital of Central South University. The patients were scanned at baseline and after 4 weeks of treatment. HCs underwent a single scan at baseline. Participants were instructed to lie supine, maintain wakefulness, keep their eyes closed, and minimize movements. Noise reduction measures included soundproof headphones and sponge earplugs during the scanning process. The scanning parameters for the resting‐state functional imaging data were as follows: repetition time (TR)/echo time (ET) = 2000 ms/30 ms; flip angle = 90°; field of view (FOV) = 220 mm; matrix = 64 × 64; slice thickness = 4 mm; number of slices = 33; gap = 0.4 mm.

The resting‐state fMRI data were preprocessed using the Data Processing Assistant for Resting‐State fMRI (DPARSF). The first 10 images were discarded to account for signal equilibration effects and to allow participants to acclimate to the scanning environment. Subsequent preprocessing steps included slice‐timing correction and head motion correction. All participants exhibited less than 2 mm of translational movement and less than 2° of rotational movement in the x, y, or z axes. Using a template generated by DARTEL, the functional images were spatially normalized to the standard MNI EPI template, resampled to a resolution of 3 mm × 3 mm × 3 mm, and smoothed using a Gaussian kernel with a full‐width at half‐maximum (FWHM) of 4 mm. The data were then linearly detrended and subjected to a temporal band‐pass filter (0.01–0.08 Hz) to mitigate the impact of low‐frequency drifts and high‐frequency physiological noise. Several covariates were regressed out, including signals from a ventricular region of interest, a region centered in the white matter, cerebrospinal fluid signals, and Friston‐24 head motion parameters obtained through rigid body correction. In accordance with a prior study,^14^ the global signal was not removed. In addition, mean framewise displacement (FD) was incorporated as a covariate in group analyses to account for residual motion effects. An aggressive motion correction strategy was also employed, involving the removal of time points with an FD greater than 0.2 mm.

**FIGURE 1:**
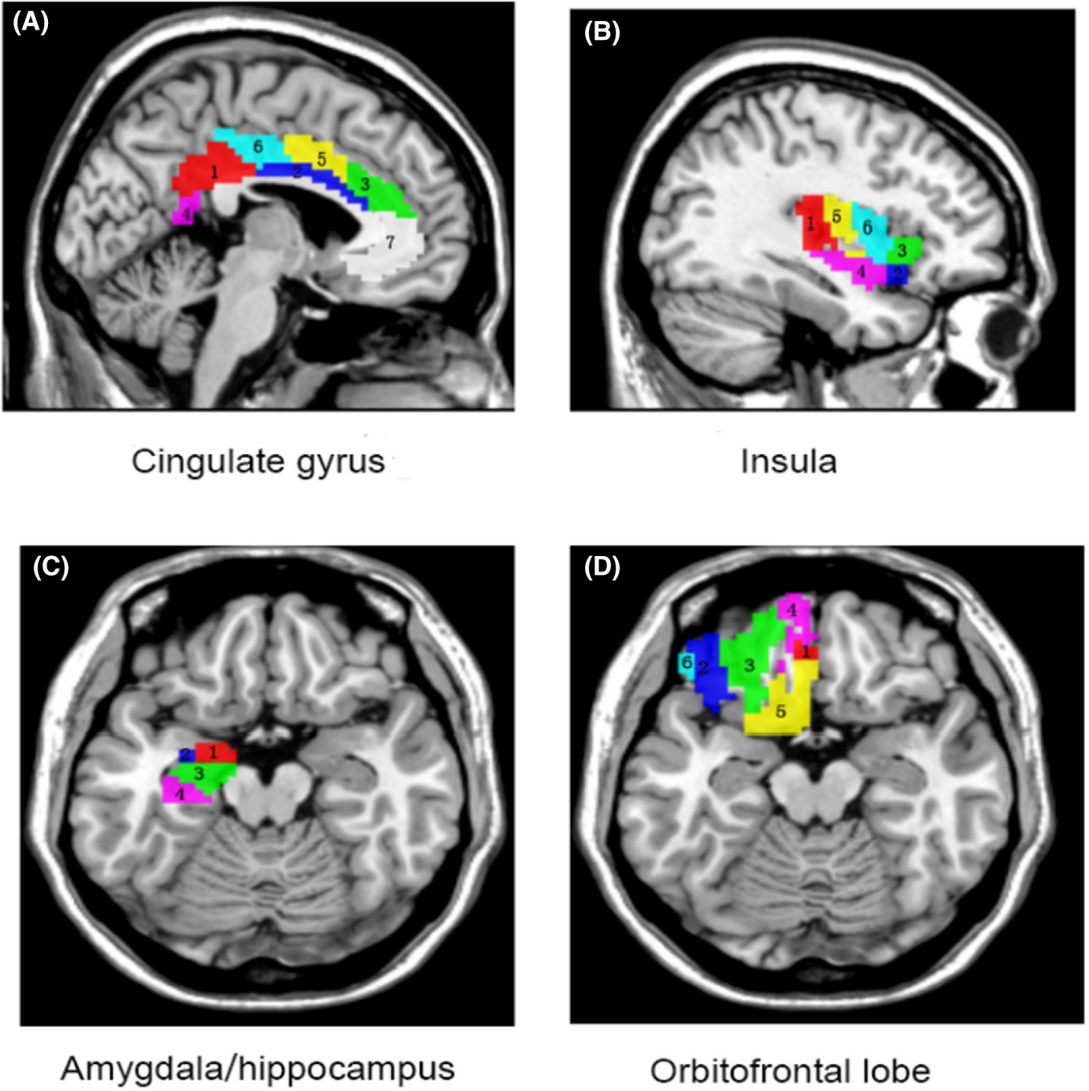
The masks of the subregions in the amygdala, cingulate gyrus, hippocampus, insula, and orbitofrontal lobe (Left side). (A)1 = dorsal area 23 (A23d); 2 = rostroventral area 24 (A24rv); 3 = pregenual area 32 (A32p); 4 = ventral area 23 (A23v); 5 = caudodorsal area 24 (A24cd); 6 = caudal area 23 (A23c); 7 = subgenual area 32 (A32sg). (B)1 = hypergranular insula (hyperG); 2 = ventral agranular insula (vIa); 3 = dorsal agranular insula (dIa); 4 = ventral dysgranular and granular insula (vId/vIg); 5 = dorsal granular insula (dIg); 6 = dorsal dysgranular insula (dId). (C) 1 = medial amygdala (mAmyg); 2 = lateral amygdala (lAmyg); 3 = rostral hippocampus (rHipp); 4 = caudal hippocampus (cHipp). (D) 1 = medial area 14 (A14m); 2 = orbital area 12/47 (A12/47o); 3 = lateral area 11 (A11l); 4 = medial area 11 (A11m); 5 = area 13 (A13); 6 = lateral area 12/47 (A12/47 L).

Using masks from the human Brainnetome atlas (http://atlas.brainnetome.org),^15^ mean fALFF values in the amygdala, cingulate gyrus, insula, hippocampus, and orbitofrontal lobe subregions were extracted (Figure 1). Subsequently, the extracted fALFF values from bilateral subregions of these brain areas for each participant were incorporated into SPSS for further statistical analysis.

As a subregion of the prefrontal cortex, the orbitofrontal cortex (OFC) plays a crucial role in guiding reward‐related behavior, decision‐making, as well as behavioral and emotional responses.^16^ The OFC maintains direct reciprocal connections with the amygdala, and existing evidence suggests that the amygdala‐OFC circuitry is integral to the neuroanatomical pathways implicated in anxiety disorders.^17^ As a result, impaired OFC function could give rise to a spectrum of behavioral deficits and psychopathological outcomes, ranging from poor financial decisions to heightened anxiety. Our focus on the orbitofrontal cortex is informed by its well‐established relevance in the context of anxiety disorders, a finding that is supported by both animal and clinical studies,^16^ as well as an extensive body of neuroimaging research.^18^

### 2.3 fALFF analysis

The DPARSF software was utilized for the fALFF calculation. The voxel time series were converted into the frequency domain via fast Fourier transform to obtain a power spectrum. Then, the square root of each power spectrum frequency was computed, and the range of 0.01–0.08 Hz was acquired for every voxel. The total amplitudes in this frequency range were normalized by dividing by the full frequency range. For standardization, each voxel's fALFF was divided by the mean whole‐brain signal amplitude, yielding a normalized fALFF map for each participant for further statistical analysis.

### 2.4 Statistical analysis

For the demographic characteristics across the three groups, continuous variables were assessed using the Kruskal–Wallis tests or the Mann–Whitney *U* tests as appropriate. Categorical variables were evaluated using chi‐square tests. Intra‐group comparisons at different time points were conducted using paired‐sample Wilcoxon tests.

A baseline fALFF comparison across the three groups was performed using an analysis of covariance (ANCOVA), adjusting for age, education years, and FD. Post hoc *t*‐tests were then used to identify group differences. The Bonferroni correction method was applied to the ROI analysis, setting the significance level at *p* < 0.05/46 = 0.00109. Whole‐brain analysis utilized Gaussian random field (GRF) theory for multiple comparison correction via rest software, with a significance level of *p* < 0.05 (voxel significance, *p* < 0.001; cluster significance, *p* < 0.05). Treatment effects were evaluated using the reduction ratio (RR) of the HAMA total scores, calculated as RR = (HAMAtotal_1 − HAMAtotal_2) / HAMAtotal_1, where HAMAtotal_1 refers to baseline HAMA scores and HAMAtotal_2 to scores following 4 weeks of treatment.

### 2.5 Correlation analyses

Partial correlation analyses were conducted to determine correlations between baseline abnormal fALFF and several clinical variables: HAMA total and related factor scores, HAMD related factor scores, cognitive and social function scores, and illness duration. The same analysis was conducted to determine the correlation between fALFF alterations and the change in clinical symptoms following treatment, with the analyses controlling for potential confounding factors, including age, years of education, FD, and HAMD total score. Multiple comparisons were corrected using the Bonferroni correction method.

### 2.6 SVR classification analysis

The SVR method was employed to ascertain if the extracted fALFF values in abnormal brain regions could predict therapeutic response. This analysis was run using the LIBSVM software package (https://www.csie.ntu.edu.tw/~cjlin/libsvm/) on Matlab R2013b. The SVR was applied to the extracted baseline fALFF and anxiety symptoms (RR of HAMA total score), with a primary focus on brain regions exhibiting significant fALFF changes post‐treatment. The algorithms and training sets used through SVR are presented below.

The objective of examining the multiple regression function f(x) in relation to x across the sample spectrum is to forecast the anticipated output characteristics. Previous studies have delineated the SVR equation,^19^, ^20^ and it can be summarized as follows^21^:

$$f\left(x\right)=a_{0}+\sum_{\mathrm{ij}=1}^{N}\left(a_{i}-{a_{i}}_{*}\right)\left\{ø\left(\mathrm{\chi i}\right).ø\left(\mathrm{\chi j}\right)\right\}+b\}$$

In this equation, 0 ≤ *α*_i_, *α*_i_* ≤ C, where C serves as a regularization parameter or penalty constant. This parameter C mediates the trade‐off between model simplicity and training error. The parameter alpha represents the set of Lagrange multipliers. Both parameters alpha and C have been thoroughly discussed in existing literature.^20^, ^22^ A noteworthy feature of SVR is its capacity to handle both linear and nonlinear data through the kernel function. The validity of the optimized model is subsequently confirmed during the prediction process. To fine‐tune the SVR model parameters, the cross‐validation method was employed to identify optimal parameters.^19^ The training dataset is partitioned into four equally sized subsets. One subset serves as the test set while the other three are utilized for predictor training, facilitating the identification of more optimal ε and c values. A grid search is conducted within a predefined parameter space. Ultimately, the model demonstrating the highest prediction accuracy, i.e., the lowest cross‐validation error, is selected for application.

## 3 RESULTS

### 3.1 Demographic and clinical characteristics

The baseline study encompassed 118 patients diagnosed with anxiety disorders (64 with GAD and 54 with PD), along with 61 HCs who successfully completed the initial MRI scanning. Subsequent exclusions due to excessive head movement during scanning were as follows: four from the GAD group, two from the PD group, and one from the HCs. Consequently, the final baseline statistical analysis incorporated 60 patients with GAD, 52 patients with PD, and 60 HCs. Comparative analyses revealed no significant differences across the three groups in terms of sex, age, and educational background. Likewise, cognitive function, scores from the SDSS, SCSQ, HAMD, and HAMA psychotic anxiety subscale showed no significant disparities. In contrast, the GAD group exhibited significantly lower HAMD anxiety/somatization subscale scores, HAMA total scores, and somatic anxiety subscale scores when compared to the PD group (Table 1).

**TABLE 1:** Characteristics of the subjects.

	GAD (*n* = 60)	PD (*n* = 52)	HCs (*n* = 60)	H/Z/χ^2^	*p*
Sex (male/female)	28/32	25/27	25/35	0.526	0.769
Age (years)	30.50 ± 10.20	31.94 ± 9.76	28.13 ± 8.57	3.793	0.150
Years of education (years)	13.90 ± 3.07	13.81 ± 3.61	14.47 ± 3.06	1.234	0.540
Illness duration (months)	16.80 ± 8.19	14.40 ± 9.75	‐	−2.050	0.040
HAMA total scores	16.38 ± 4.79	19.21 ± 7.86	‐	−1.980	0.048
Psychotic anxiety	10.83 ± 3.07	11.17 ± 4.26	‐	−0.346	0.729
Somatic anxiety	5.52 ± 2.83	7.96 ± 3.79	‐	−3.813	<0.001
HAMD total scores	13.68 ± 3.47	14.08 ± 2.98	‐	−0.384	0.701
Anxiety/somatization	6.18 ± 1.57	6.83 ± 1.40	‐	−2.577	0.010
Cognitive disorder	1.60 ± 1.15	1.29 ± 0.89	‐	−1.470	0.142
Retardation	3.05 ± 1.33	3.02 ± 1.43	‐	−0.180	0.858
Insomnia	2.60 ± 1.65	2.60 ± 1.54	‐	−0.027	0.979
Loss of weight	0.27 ± 0.55	0.40 ± 0.66	‐	1.394	0.241
B‐CATS			‐		
Digit symbol test	53.92 ± 13.29	51.31 ± 12.49	‐	−1.147	0.251
TMT‐A	38.15 ± 14.75	37.07 ± 13.02	‐	−0.020	0.984
TMT‐B	66.58 ± 31.16	71.35 ± 29.34	‐	−1.065	0.287
Animal naming fluency test	18.08 ± 5.83	17.83 ± 4.53	‐	−0.205	0.838
SCSQ			‐		
Active coping	20.47 ± 6.65	19.00 ± 6.75	‐	−1.005	0.315
Passive coping	9.97 ± 4.36	11.02 ± 4.13	‐	−1.390	0.164
SDSS	3.63 ± 2.65	4.33 ± 3.39	‐	−0.954	0.340

Abbreviations: B‐CATS, Brief Cognitive Assessment for Schizophrenia; HAMA, Hamilton Anxiety Scale; HAMD, Hamilton Depression Scale; SCSQ, Simple Coping Style Questionnaire; SDSS=Social Disability Screening Schedule; TMT, Trail Making Test.

### 3.2 Symptom improvement following treatment

During the follow‐up phase, 12 patients with GAD and 10 patients with PD withdrew from the study due to factors including the COVID‐19 pandemic, medication discontinuation, and loss to follow‐up. Thus, 52 patients with GAD and 44 patients with PD completed the follow‐up process. Of these remaining participants, an additional six patients with GAD and two patients with PD were excluded due to excessive head movement, yielding 46 patients in the GAD group and 42 patients in the PD group for final statistical analysis at follow‐up. Post‐treatment results, following a 4‐week period, showed significant improvements in HAMA total and subscale scores, HAMD total and subscale scores, SDSS scores, and scores on the B‐CATS within both patient groups (Table 2).

**TABLE 2:** Comparison of the clinical characteristics between the GAD group and the PD group at each time point.

	GAD (*n* = 46)		Z	*p*	PD (*n* = 42)		Z	*p*	*p* ^a^	*p* ^b^
	Baseline	4 weeks			Baseline	4 weeks				
HAMA	16.72 ± 5.15	8.98 ± 5.31	−5.372	<0.001	19.00 ± 8.34	8.14 ± 5.43	−5.649	<0.001	0.253	0.298
Psychotic anxiety	11.00 ± 3.30	5.89 ± 3.23	−5.408	<0.001	11.00 ± 4.49	4.74 ± 3.08	−5.621	<0.001	0.387	0.072
Somatic anxiety	5.67 ± 2.82	3.09 ± 2.83	−4.397	<0.001	7.90 ± 3.99	3.40 ± 2.79	−5.511	<0.001	0.003	0.440
HAMD	13.39 ± 3.63	8.37 ± 5.36	−4.639	<0.001	13.95 ± 3.11	7.45 ± 5.03	−5.176	<0.001	0.557	0.320
Anxiety/somatization	6.20 ± 1.67	3.57 ± 2.15	−4.967	<0.001	6.83 ± 1.36	3.43 ± 2.45	−5.084	<0.001	0.021	0.576
Cognitive disorder	1.39 ± 1.04	0.76 ± 1.02	−3.184	0.001	1.36 ± 0.93	0.45 ± 0.63	−4.429	<0.001	0.875	0.271
Retardation	2.87 ± 1.33	1.83 ± 1.74	−3.684	<0.001	2.83 ± 1.36	1.50 ± 1.71	−4.242	<0.001	0.976	0.283
Insomnia	2.70 ± 1.68	2.07 ± 1.82	−2.620	0.009	2.62 ± 1.53	1.95 ± 1.37	−2.572	0.010	0.829	0.885
Loss of weight	0.26 ± 0.53	0.15 ± 0.36	−1.107	0.268	0.38 ± 0.66	0.12 ± 0.40	7.328	0.010	0.414	0.453
B‐CATS										
Digit symbol test	51.98 ± 13.47	57.02 ± 13.53	−5.159	<0.001	50.10 ± 13.09	55.19 ± 13.96	−3.987	<0.001	0.432	0.496
TMT‐A	39.28 ± 15.93	32.29 ± 19.23	−4.283	<0.001	38.51 ± 13.62	33.52 ± 11.66	−2.663	0.008	0.767	0.150
TMT‐B	67.92 ± 33.69	57.62 ± 37.31	−3.704	<0.001	72.93 ± 30.39	62.50 ± 26.41	−3.164	0.002	0.310	0.080
Animal naming fluency test	17.24 ± 5.40	18.87 ± 5.54	−2.381	0.017	17.31 ± 4.62	18.24 ± 4.49	−1.539	0.124	0.930	0.815
SCSQ										
Active coping	20.98 ± 6.78.	19.97 ± 9.21	−0.320	0.749	18.62 ± 7.15	20.40 ± 8.35	−1.926	0.054	0.140	0.917
Passive coping	9.93 ± 4.35	9.13 ± 5.05	−0.526	0.599	10.86 ± 4.11	10.07 ± 4.97	−0.782	0.435	0.202	0.393
SDSS	3.52 ± 2.76	2.13 ± 3.00	−3.201	0.001	4.36 ± 3.53	2.00 ± 2.55	−4.598	<0.001	0.340	0.906

*Note*: *p* values was obtained within group from baseline (time point 1) to treatment (time point 2) by Pairs Sample Wilcoxon test. *p* ^a^: *p* value was obtained with comparison between two groups at baseline (time point 1) by the Mann–Whitney *U* test. *p* ^b^: *p* value was obtained with comparison between two groups after treatment (time point 2) by the Mann–Whitney *U* test.

### 3.3 Group differences in fALFF at baseline

**FIGURE 2:**
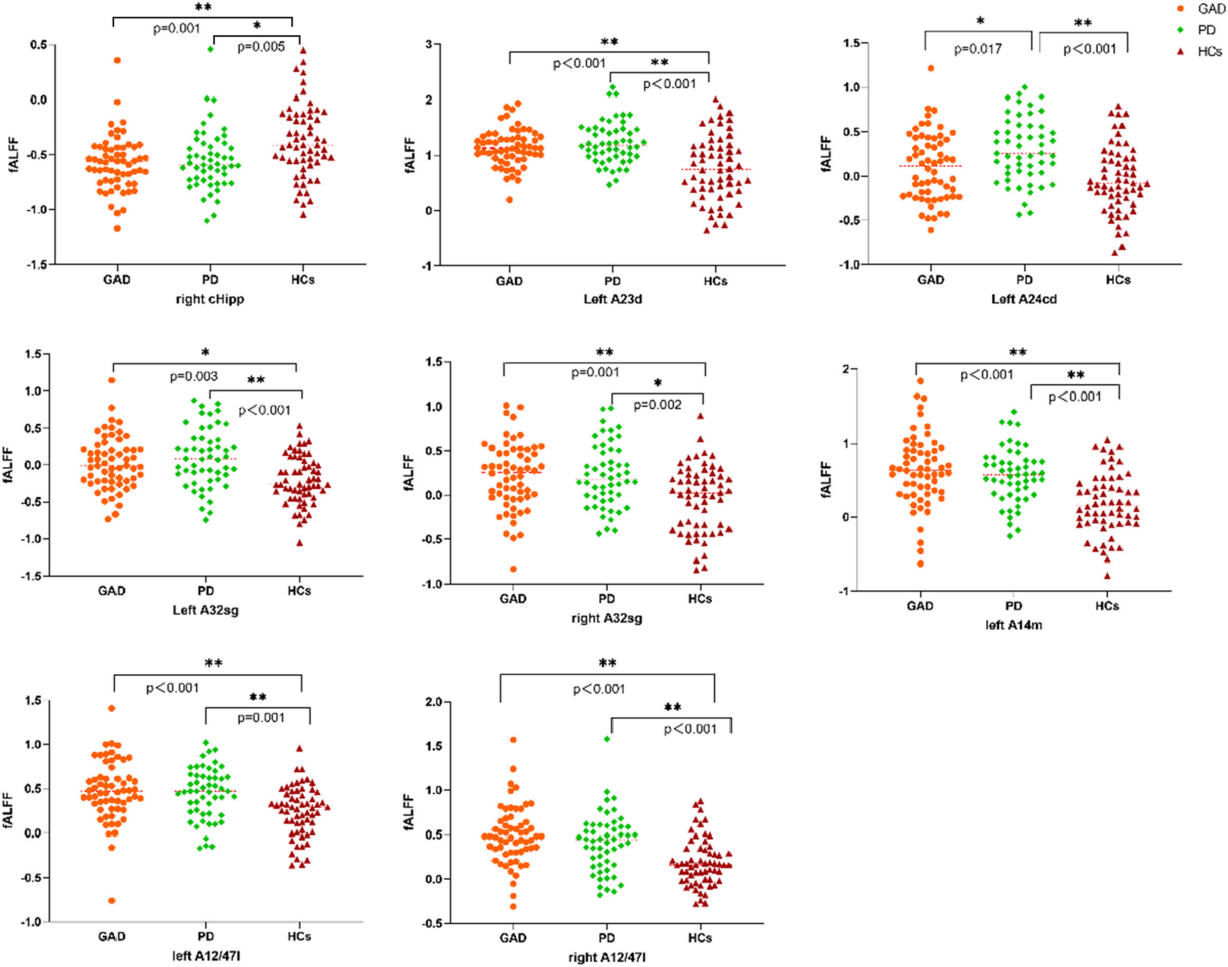
fALFF comparison in subregions. A12/47L, lateral area 12/47; A14m, medial area 14; A23d, dorsal area 23; A24cd, caudodorsal area 24; A32sg, subgenual area 32; cHipp, caudal hippocampus; fALFF, fractional Amplitude of Low Frequency Fluctuation.

Utilizing a Bonferroni correction at the 0.05/46 = 0.00109 level, discernable variations in fALFF values were noted across three distinct groups within the right cHipp subregion of the hippocampus, left A23d, A24cd, bilateral A32sg subregions of the cingulate gyrus, and left A14m alongside bilateral A12/47l subregions of the orbitofrontal lobe. Supplementary post hoc *t*‐tests delineated substantial reductions in fALFF within the right cHipp subregion of the hippocampus in both GAD (*p* = 0.001) and PD (*p* = 0.005) patients compared to HCs. In contrast, the fALFF values in the left A24cd subregion of the cingulate gyrus in patients with PD exceeded those in patients with GAD and HCs, while no significant differences were detected between patients with GAD and HCs in the same region. Conversely, within the left A23d, bilateral A32sg of the cingulate gyrus, and aforementioned orbitofrontal lobe subregions, patients with GAD and PD displayed elevated fALFF values relative to HCs, with no significant differences discerned between the two patient groups (Figure 2). No statistically significant variations in fALFF values were found in the bilateral amygdala, insula, and other subregions of the ROI across all three groups (Table 3).

**TABLE 3:** Group comparison of fALFF in different subregions at baseline.

	L/R	GAD (*n* = 60)	PD (*n* = 52)	HCs(*n* = 60)	F	Uncorrected *p*	Post hoc *t*‐tests
Amygdala							
mAmyg	L	−0.5343 ± 0.5238	−0.5383 ± 0.5463	−0.4564 ± 0.32288	0.901	0.408	
mAmyg	R	−0.4863 ± 0.5198	−0.4262 ± 0.3998	−0.5397 ± 0.4827	0.485	0.616	
lAmyg	L	−0.4400 ± 0.3316	−0.3992 ± 0.3489	−0.4033 ± 0.3655	0.293	0.746	
lAmyg	R	−0.4249 ± 0.4096	−0.3347 ± 0.4116	−0.3677 ± 0.3746	0.622	0.538	
Hippocampus							
rHipp	L	−0.5092 ± 0.3326	−0.4739 ± 0.3092	−0.5130 ± 0.2911	0.116	0.890	
rHipp	R	−0.5053 ± 0.3781	−0.4017 ± 0.3218	−0.4796 ± 0.3630	1.093	0.338	
cHipp	L	−0.5515 ± 0.2304	−0.5859 ± 0.2079	−0.4103 ± 0.4145	6.482	0.002	
cHipp	R	−0.5786 ± 0.2477	−0.5495 ± 0.2736	−0.3760 ± 0.3322	8.567	0.000288	GAD<HCs, PD<HCs
Insula							
hyperG	L	−0.3782 ± 0.2763	−0.2871 ± 0.2818	−0.1537 ± 0.4550	5.970	0.003	
hyperG	R	−0.3418 ± 0.3128	−0.2153 ± 0.3354	−0.1899 ± 0.4202	2.932	0.056	
vIa	L	−0.3879 ± 0.2966	−0.3199 ± 0.2982	−0.2962 ± 0.4476	0.884	0.415	
vIa	R	−0.3532 ± 0.2994	−0.2198 ± 0.3474	−0.2734 ± 0.4013	2.105	0.125	
dIa	L	−0.1183 ± 0.3565	−0.0419 ± 0.3399	−0.0631 ± 0.3076	0.821	0.442	
dIa	R	0.0566 ± 0.3882	0.1224 ± 0.3623	0.0123 ± 0.3478	0.793	0.454	
vId/vIg	L	−0.3212 ± 0.4150	−0.2617 ± 0.3449	−0.2786 ± 0.4082	0.333	0.717	
vId/vIg	R	−0.2776 ± 0.4083	−0.2293 ± 0.3594	−0.1968 ± 0.3537	0.592	0.554	
dIg	L	−0.3187 ± 0.3046	−0.2388 ± 0.3242	−0.1331 ± 0.4505	3.396	0.036	
dIg	R	−0.2475 ± 0.2790	−0.1670 ± 0.3112	−0.1027 ± 0.3968	2.781	0.065	
dId	L	−0.2127 ± 0.3031	−0.1218 ± 0.3709	−0.1054 ± 0.3739	1.539	0.218	
dId	R	−0.2076 ± 0.2925	−0.0297 ± 0.3684	−0.1069 ± 0.3157	4.058	0.019	
Cingulate gyrus							
A23d	L	1.1431 ± 0.3333	1.2270 ± 0.3833	0.7698 ± 0.5920	17.142	<0.0001	GAD>HCs, PD>HCs
A23d	R	0.9567 ± 0.3343	1.0950 ± 0.3930	0.8837 ± 0.6378	2.954	0.055	
A24rv	L	0.0895 ± 0.4368	0.1527 ± 0.4511	−0.1131 ± 0.4105	5.621	0.004	
A24rv	R	−0.3555 ± 0.3688	−0.2315 ± 0.3760	−0.3140 ± 0.3184	1.730	0.180	
A32p	L	0.0903 ± 0.2402	0.1221 ± 0.2948	−0.0445 ± 0.3424	5.034	0.008	
A32p	R	0.3943 ± 0.3697	0.4082 ± 0.3137	0.2364 ± 0.4297	3.911	0.022	
A23v	L	0.4935 ± 0.3246	0.4546 ± 0.3201	0.5539 ± 0.5436	0.912	0.404	
A23v	R	0.4177 ± 0.3801	0.5259 ± 0.4082	0.7208 ± 0.5388	6.392	0.002	
A24cd	L	0.1007 ± 0.3803	0.2984 ± 0.3611	−0.0332 ± 0.3795	10.798	0.000039	GAD<PD, PD>HCs
A24cd	R	0.2009 ± 0.4665	0.3312 ± 0.4283	0.2404 ± 0.3964	1.227	0.296	
A23c	L	−0.0072 ± 0.2919	0.0647 ± 0.3241	0.0439 ± 0.3993	0.675	0.511	
A23c	R	0.1644 ± 0.3055	0.3373 ± 0.3266	0.3771 ± 0.4517	5.569	0.005	
A32sg	L	0.0283 ± 0.3588	0.0966 ± 0.3976	−0.2025 ± 0.3280	10.042	0.000076	GAD>HCs, PD>HCs
A32sg	R	0.2135 ± 0.3803	0.2153 ± 0.3564	−0.0373 ± 0.3758	8.275	0.000375	GAD>HCs, PD>HCs
Orbitofrontal lobe							
A14m	L	0.6362 ± 0.4850	0.5664 ± 0.3750	0.1889 ± 0.4136	18.110	<0.0001	GAD>HCs, PD>HCs
A14m	R	0.3481 ± 0.3980	0.2838 ± 0.3600	0.0850 ± 0.4340	6.544	0.002	
A12/47o	L	0.1308 ± 0.3150	0.0493 ± 0.2850	−0.0488 ± 0.2710	5.769	0.004	
A12/47o	R	0.2357 ± 0.3932	0.1990 ± 0.4256	0.0492 ± 0.3227	4.006	0.020	
A11l	L	−0.1196 ± 0.3450	−0.1997 ± 0.3330	−0.2208 ± 0.3300	1.357	0.260	
A11l	R	−0.0554 ± 0.3413	−0.1175 ± 0.3090	−0.2457 ± 0.3120	4.475	0.013	
A11m	L	0.1062 ± 0.4406	0.0975 ± 0.3795	−0.1583 ± 0.4370	6.673	0.002	
A11m	R	0.2641 ± 0.3632	0.2126 ± 0.3679	0.0525 ± 0.4407	4.500	0.013	
A13	L	−0.5097 ± 0.3419	−0.5454 ± 0.3750	−0.5759 ± 0.3590	0.353	0.703	
A13	R	−0.4719 ± 0.3622	−0.4680 ± 0.3840	−0.6312 ± 0.4100	2.240	0.110	
A12/47 L	L	0.4776 ± 0.3380	0.4473 ± 0.2880	0.2451 ± 0.2810	10.685	0.000043	GAD>HCs, PD>HCs
A12/47 L	R	0.4890 ± 0.3255	0.4075 ± 0.3302	0.1911 ± 0.2688	16.265	<0.0001	GAD>HCs, PD>HCs

Abbreviations: A11l, lateral area 11; A11m, medial area 11; A12/47 L, lateral area 12/47; A12/47o, orbital area 12/47; A13, area 13; A14m, medial area 14; A23c, caudal area 23; A23d, dorsal area 23; A23v, ventral area 23; A24cd, caudodorsal area 24; A24rv, rostroventral area 24; A32p, pregenual area 32; A32sg, subgenual area 32; cHipp, caudal hippocampus; dIa, dorsal agranular insula; dId, dorsal dysgranular insula; dIg, dorsal granular insula; hyperG, hypergranular insula; lAmyg, lateral amygdala; mAmyg, medial amygdala; rHipp, rostral hippocampus; vIa, ventral agranular insula; vId/vIg, ventral dysgranular and granular insula.

A whole‐brain ANCOVA identified significant differences in fALFF values across the groups within the cingulate gyrus (including both anterior and posterior cingulate gyrus), inferior orbitofrontal gyrus (OFG), middle frontal gyrus, and medial OFG. Further post hoc *t*‐tests showed that elevated fALFF values were observed within the medial OFG/anterior and paracingulate gyrus, medial posterior/paracingulate, inferior OFG/middle OFG, and anterior and paracingulate gyrus in patients with GAD and PD when compared with HCs, further validating functional deficits in the cingulate gyrus and orbitalfrontal lobe identified through whole‐brain analysis (Table S1; Figure S1). Notably, no substantial differences were found in the fALFF values between the PD and GAD groups.

### 3.4 fALFF alterations in patient groups following treatment

**FIGURE 3:**
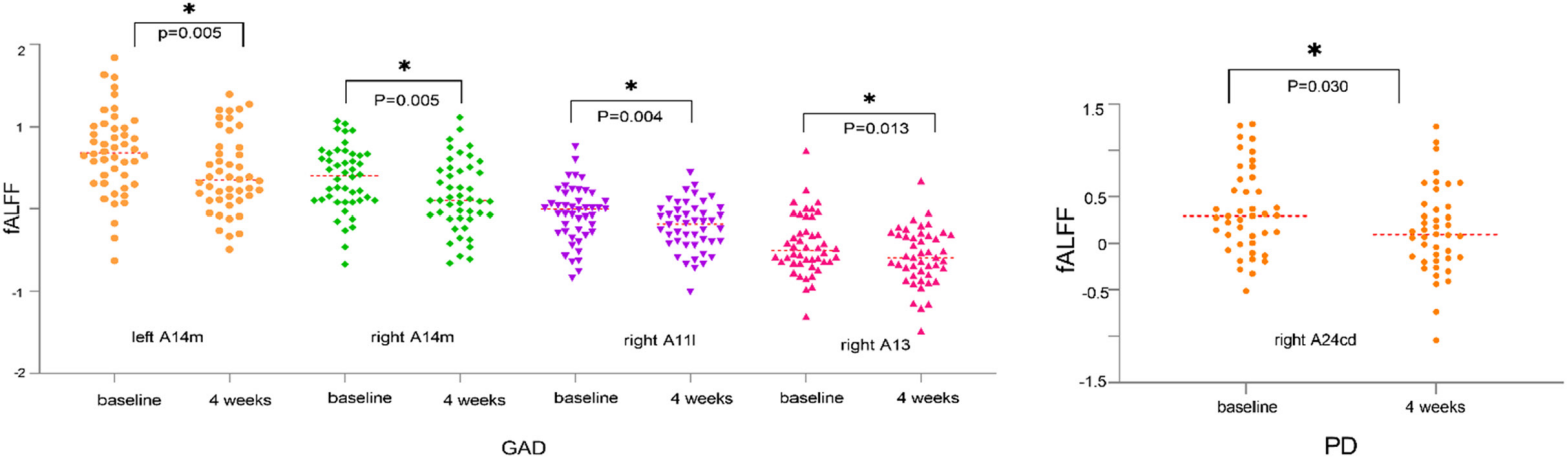
fALLF changes after 4 weeks of treatment within groups.

Upon the completion of a 4‐week treatment, patients with GAD exhibited significant diminution in fALFF values within the bilateral A14m, right A11l, and A13 subregions of the orbitofrontal lobe (*p* < 0.05). Similarly, fALFF values in the right A24cd subregion of the cingulate gyrus markedly decreased in patients with PD post‐treatment (Figure 3). However, other ROI regions in both patient groups showed no significant post‐treatment alterations in fALFF values.

The whole‐brain analysis revealed a reduction in fALFF values in the right superior OFG/inferior OFG in the GAD group following a 4‐week treatment regimen. This finding further corroborates the post‐treatment alterations in functional activity within the orbitofrontal lobe, as evidenced by whole‐brain analysis (Table S2). Conversely, no significant modifications were observed in the fALFF values for patients with PD after 4 weeks of treatment.

### 3.5 Correlation analysis

**FIGURE 4:**
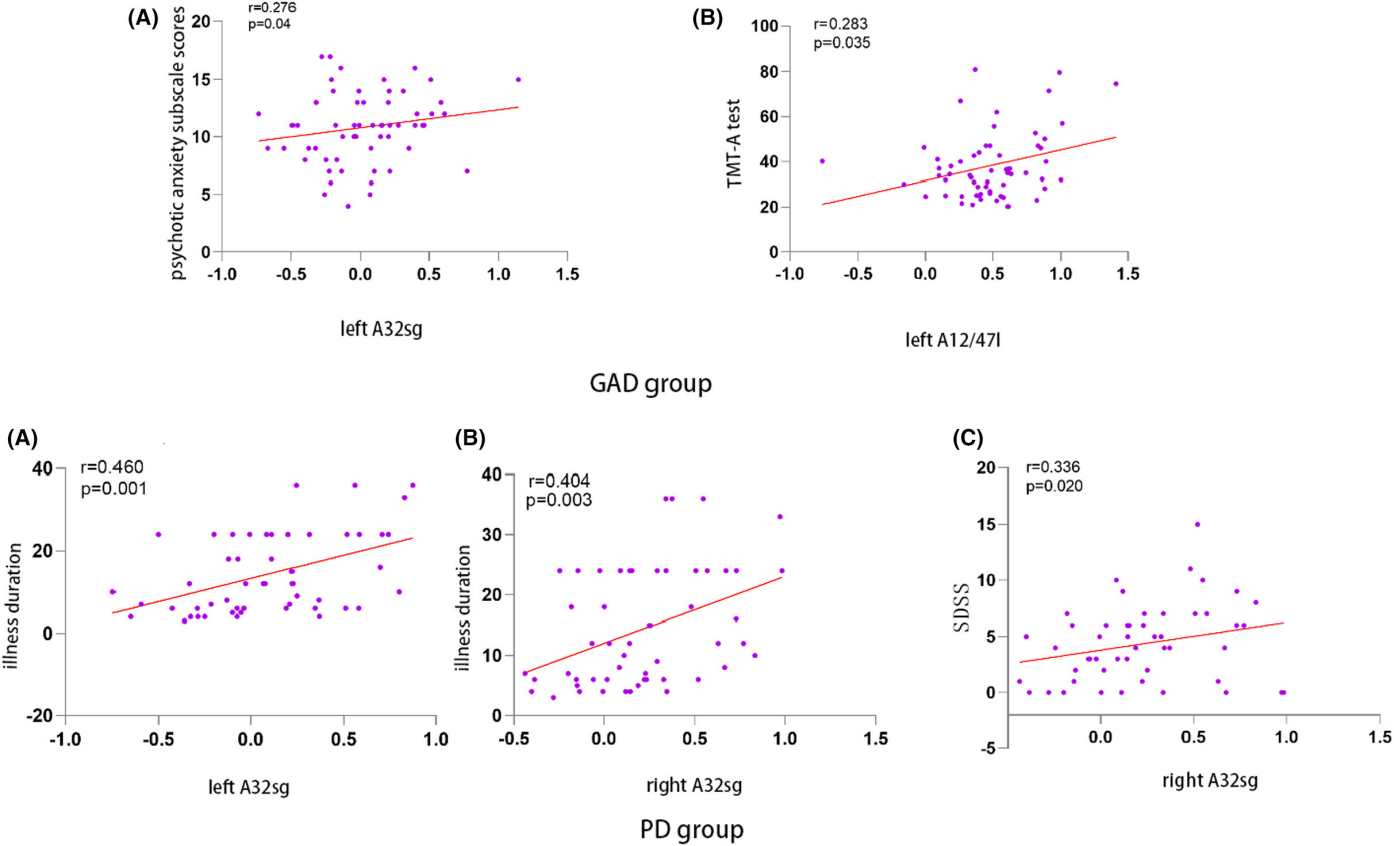
Correlations between abnormal fALFF and clinical indicators in patient groups. In the GAD group, the fALFF in the left A32sg subregion of the cingulate gyrus was positively correlated with psychotic anxiety subscale scores (*r* = 0.276, *p* = 0.04). The left fALFF in the A12/47 L subregion of the orbitofrontal lobe was positively correlated with TMT‐A (*r* = 0.283, *p* = 0.035). In PD group, the fALFF in the bilateral A32sg subregion of the cingulate gyrus was positively correlated with the illness duration. The fALFF in the right A32sg subregion of the cingulate gyrus was positively correlated with SDSS (a, *r* = 0.460, *p* = 0.001; b, *r* = 0.404, *p* = 0.003; c, *r* = 0.336, *p* = 0.020).

Within the GAD cohort, a positive correlation was found between fALFF values in the left A32sg subregion of the cingulate gyrus and HAMA psychotic anxiety subscale scores (*r* = 0.276, *p* = 0.04). Also, the left A12/47l subregion of the orbitofrontal lobe demonstrated a positive correlation between fALFF values and the TMT‐A test (*r =* 0.283, *p* = 0.035). In the PD cohort, fALFF values in the bilateral A32sg subregions of the cingulate gyrus positively correlated with the duration of the illness (left: *r* = 0.460, *p* = 0.001; right: *r* = 0.404, *p* = 0.003). Furthermore, fALFF values in the right A32sg subregion demonstrated a positive correlation with SDSS (*r* = 0.336, *p* = 0.020) (Figure 4). However, none of these correlations remained significant following Bonferroni multiple comparisons (*p* < 0.05/13 × 8). No significant correlations were identified between fALFF changes and clinical indicators' alterations, such as cognitive function and HAMA total scores after 4 weeks of treatment.

### 3.6 SVR analysis

**FIGURE 5:**
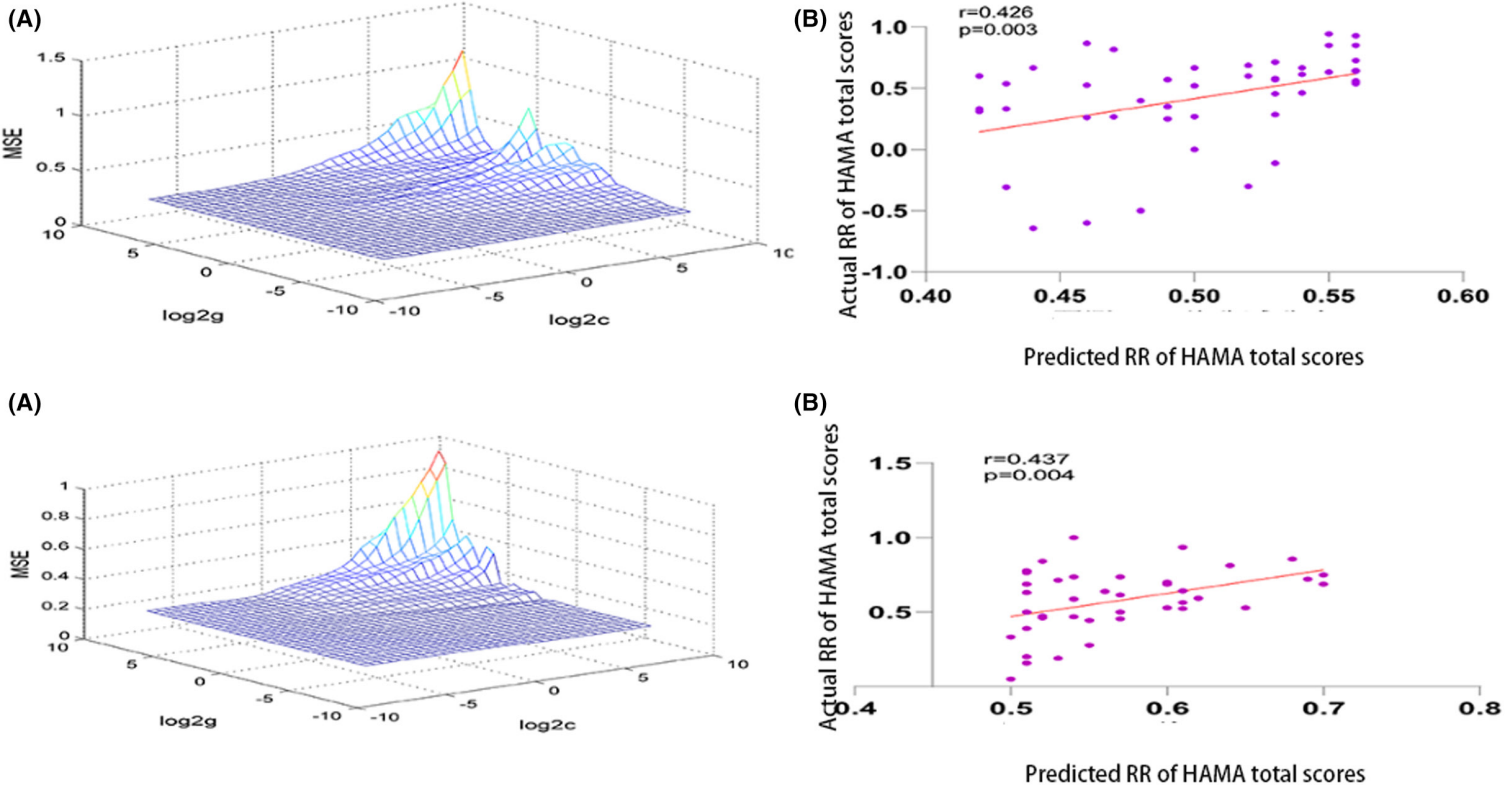
Increased fALFF levels in the right A13 subregion of the orbitofrontal lobe in the GAD group predicted treatment response. The increased fALFF level in the right A24cd subregion of the cingulate gyrus in PD group could predict the treatment response. (A) 3D visualization of SVR (results of optimal parameter selection); (B) the predicted RR of HAMA total scores were positively correlated with the actual RR. RR, reduction rate.

Based on our hypotheses and treatment outcomes, we directed further SVR investigations toward key anatomical regions: right A24cd, bilateral A14m, right A11l, and A13 subregions. Upon applying a Bonferroni correction at a significance level of 0.01 (0.05/5), we discerned significant correlations in the GAD group between the predicted and actual symptom improvements within the right A13 subregion of the orbitofrontal lobe. This finding was based on the RR of HAMA total scores (*r* = 0.426, *p* = 0.003) (Figure 5). Concurrently, a similar correlation was observed in the PD group, specifically within the right A24cd subregion of the cingulate gyrus (*r* = 0.437, *p* = 0.004).

The SVR findings indicated that elevated fALFF values, when contrasted with HCs, within these specific brain subregions were predictive of more favorable treatment outcomes for both patient groups. In order to corroborate these findings, we stratified each patient group into high and low RR subsets, using the median RR of HAMA total scores as a divider. Within the GAD group, patients in the high RR subset exhibited higher fALFF values within the right A13 subregion of the orbitofrontal lobe than those in the low RR subset (high RR: −0.4170 ± 0.3430; low RR: −0.4621 ± 0.3900). Similarly, the PD high RR subset exhibited higher fALFF values within the right A24cd subregion of the cingulate gyrus than the low RR subset (high RR: 0.4217 ± 0.5136; low RR: 0.2750 ± 0.3961). Despite these differences failing to achieve statistical significance (GAD: *t* = ‐0.417, *p* = 0.678; PD: *t* = ‐1.036, *p* = 0.306), the fALFF values in both high RR groups exhibited an increasing trend.

## 4 DISCUSSION

The findings of this study demonstrate a notable decrease in the fALFF values within the right cHipp subregion of the hippocampus, whereas the left areas A23d, A24cd, and bilateral A32sg subregions of the cingulate gyrus, as well as the left A14m and bilateral A12/47l subregions of the orbitofrontal lobe, exhibited increased fALFF values in both GAD and PD groups when compared to HCs at baseline. In patients with PD, the fALFF value in the left A24cd subregion was notably higher than that in the GAD group. These observations were further reinforced by a whole‐brain analysis, which identified aberrant fALFF in the anterior and posterior cingulate, collateral cingulate gyrus, inferior OFG, middle frontal gyrus, and medial OFG in patients with GAD and PD in contrast with HCs, confirming our ROI results. Following 4 weeks of treatment, the fALFF values in the bilateral A14m, right A11l, and A13 subregions of the orbitofrontal lobe significantly decreased within the GAD group. Concurrently, the PD group exhibited a significant decrease in fALFF values in the right A24cd subregion of the cingulate gyrus. In addition, the SVR analysis indicates that the fALFF in the right A13 and right A24cd subregions might be effective predictors of treatment response in patients with anxiety disorders.

The cingulate cortex, a prominent component of the limbic system, plays an important role in regulating emotions, driving behavioral learning, and supporting memory.^23^ Prior neuroimaging studies have evidenced the participation of the ACC and the posterior cingulate cortex in the neuropathological mechanisms of anxiety disorders.^24^, ^25^ Nonetheless, studies on functional activity in the cingulate gyrus in the context of anxiety disorders have yielded inconsistent results. For instance, task‐state studies indicated heightened neural activation of the posterior cingulate and ACC in patients with PD when exposed to panic stimuli.^26^ Meanwhile, a resting‐state study by Wang et al. demonstrated elevated ALFF in the left precuneus/posterior cingulate gyrus in patients with GAD, revealing a negative correlation between the FC of this region and the left ACC and anxiety symptomatology.^27^ However, the majority of these studies did not examine the subregions within the cingulate gyrus, a factor that could contribute to the observed inconsistencies. The divergence in results may stem from the distinct functions attributable to the various regions within the cingulate cortex.^23^ Specifically, the ACC integrates information related to mood and reward from structures such as the orbitofrontal lobe and amygdala, and relays it to several brain regions, including the brain stem and insula. Conversely, the posterior cingulate gyrus, receiving primary input from the precuneus and parietal cortical regions, is involved in spatial processing, spatial action, and some forms of memory.^28^ Consequently, investigating the individual regions of the cingulate gyrus can enhance our understanding of its specific role in the pathophysiology of anxiety disorders. In our study, we partitioned the cingulate gyrus into 14 subregions using a robustly cross‐validated and well‐defined brain atlas, thus reducing its inherent heterogeneity. We observed elevated fALFF values in the A23d and bilateral A32sg subregions in both patients with GAD and PD compared to HCs, aligning with our hypothesis. This increased functional activity in these subregions could represent a common neuropathological mechanism for anxiety disorders. Interestingly, the fALFF values in the left A24cd subregion were significantly higher in patients with PD than in patients with GAD, while no significant variations were detected in the other subregions. Hence, we posit that the functional activity in the left A24cd subregion may be associated with specific neurobiological alterations in different anxiety disorder subtypes.

Our study also revealed reduced fALFF values within the right cHipp subregion of the hippocampus in patients diagnosed with both GAD and PD. Previous studies have identified the hippocampus as central to situational fear learning, serving to mediate responses to external stimuli and playing an integral role in the confluence of emotion and cognition.^29^, ^30^ Consequently, any malfunction within the hippocampus can disrupt its interaction with the PFC and other components of the limbic system. Moon et al. discovered a decrease in both activity and gray matter volume within the hippocampal region of patients with GAD compared to HCs.^31^ Meanwhile, Shen et al. found elevated ALFF in the left hippocampal region of patients with GAD who responded positively to treatment as opposed to non‐responders, suggesting enhanced hippocampal functional activity might be conducive to symptom remission.^32^ Our ROI analysis detected a reduction in functional activity within the right cHipp subregion of the hippocampus in patients with anxiety disorders, corroborating prior findings. Therefore, we postulate that diminished fALFF within the hippocampus could underpin the neural basis for the attenuation of fear and environmental learning disabilities observed in patients with anxiety disorders.

We centered our investigation on the ventromedial prefrontal lobe, particularly the orbitofrontal lobe, due to its established significance in the context of anxiety disorders,^16^ a conclusion corroborated by numerous neuroimaging studies.^18^ Our data unveiled heightened fALFF values within the left A14m and bilateral A12/47l subregions in both patients with GAD and PD. Our whole‐brain analysis identified similar increases in functional activity within the orbitofrontal lobe. This hyperactivation has been linked to self‐referential and higher evaluative processes.^33^ Prior studies indicate a positive correlation between the neural activity of the right OFG and illness duration, implying that hyperactivation of the OFG may signify prolonged illness duration in patients with GAD.^32^ A resting‐state study reported increased ALFF within the superior OFG and right OFG in patients with anxiety disorders.^34^ These findings suggest that regional activity anomalies within specific subregions of the orbitofrontal lobe could denote dysfunctional cortical regions that have a direct bearing on the manifestation of anxiety disorders.

No significant differences were detected in the fALFF values within the amygdala and insular subregions across the three groups. Several factors may account for this. First, given their small size and deep brain location, further subdivision of the amygdala and insula might yield relatively small voxels, which could obscure noticeable differences. Second, abnormal activity within these regions primarily manifests during task‐related studies, suggesting that the amygdala and insula might be less reactive during spontaneous brain activities. For instance, a study by Lai et al.^35^ did not report differences in functional brain activities of the amygdala and insula during resting state. Last, the limited signal‐to‐noise ratio of functional MRI within the basal ganglia region and deep lateral fissure during resting state may be a contributing factor.

Following 4 weeks of treatment, significant reductions in fALFF values were observed in the bilateral A14m, right A11l, and right A13 subregions in the GAD group. Furthermore, fALFF values in the right A24cd subregion markedly decreased within the PD group. These alterations suggest a trend toward normalization of brain functional activities within these subregions, potentially benefiting from the effects of antidepressant therapy. Notably, changes within single right A24cd subregion through ROI analysis in PD group might not survive multiple comparison corrections in the whole‐brain analysis. Previous studies, such as Martin et al.,^36^ have suggested that antidepressant treatments augment local cerebral blood flow, while Drevets et al.^37^ have found normalization of blood flow within certain cerebral structures post‐antidepressant therapy. These mechanisms could provide insight into the association between paroxetine treatment and enhancements in specific subregions of the orbitofrontal lobe and cingulate gyrus. However, we observed that the majority of these subregions exhibiting differences following treatment were not abnormal at baseline. Conversely, some brain regions with abnormal spontaneous activity at baseline did not demonstrate recovery following treatment. We hypothesize that a short‐term, 4‐week drug regimen may be inadequate to induce statistically significant changes in functional activity recovery. In addition, it is plausible that these subregions, which show significant differences post‐treatment, may serve as potential predictors of drug treatment response and are inherently sensitive to pharmacological intervention. Further analysis using SVR indicated that elevated baseline fALFF values in the right A13 and right A24cd subregions might serve as predictors for treatment response. This underlines the significance of these specific orbitofrontal and cingulate gyrus subregions in anxiety disorders and could inform potential targets for future therapeutic interventions.

This study does present several limitations. First, despite our efforts to mitigate the impact of depressive symptoms through strict inclusion criteria and the inclusion of these symptoms as covariates, it is possible that the effects of depression were not entirely eliminated due to the high‐comorbidity rate with anxiety disorders. Second, we only assessed the effects of paroxetine treatment over a 4‐week period, hence, the long‐term impacts of this therapy remain unexplored. Third, the absence of longitudinal data for the HCs group limits the study's capacity for a repeated‐measures comparison with the patient cohort. Consequently, attributing the observed changes in the patients solely to the intervention becomes challenging. Nonetheless, prior research has indicated that fALFF serves as a relatively stable metric in resting‐state fMRI studies.^38^ In HCs, minimal intra‐individual variations between the baseline (t1) and final (t2) scans may be expected, as measured by fMRI. In addition, the emotional state of the patients, such as anxiety or other negative emotions, may affect the results during MR scans. For example, the emotional profile of patients experiencing negative emotions diverges from that of individuals who are simply inattentive. Therefore, the study's findings warrant cautious interpretation.

In conclusion, this investigation represents the inaugural study examining regional activity within the prefrontal‐limbic system circuitry using a finely tuned Brainnetome atlas. This method aids in reducing the heterogeneity of brain regions under scrutiny. The evidence indicates that abnormal regional activity in the right cHipp subregion of the hippocampus, left A23d and bilateral A32sg subregions of the cingulate, as well as the left A14m and bilateral A12/47l subregions of the orbitofrontal lobe, may be related to anxiety‐induced state changes. In addition, high fALFF levels in the right A13 and right A24cd subregions could serve as predictors for individual responses to antipsychotic therapy, suggesting these subregions as potential targets for future anxiety disorder treatments. Consequently, this study provides new insights into abnormal regional activity while providing more precise anatomical localization information for those living with anxiety disorders.

## AUTHOR CONTRIBUTIONS

All the authors contributed to and approved the final manuscript. Wenbin Guo, Xiaoxiao Shan Jingping Zhao designed the study. Xiaoxiao Shan, Haohao Yan, Huabing Li, Ping Li collected the original imaging data. Wenbin Guo, Feng Liu managed and analyzed the imaging data, and Xiaoxiao Shan wrote the first draft of the manuscript.

## FUNDING INFORMATION

This study was supported by grants from the National Natural Science Foundation of China (Grant Nos. 82171508) and Natural Science Foundation of Hunan (Grant No. 2020JJ4784).

## CONFLICT OF INTEREST STATEMENT

We declare that there is no any potential interest conflict for this study.

## Supporting information


Figure S1.

Table S1.
Table S2.
Table S3.

## Data Availability

The data that support the findings of this study are available from the corresponding author upon reasonable request.
