# Supplementary figures and images for: Abnormal regional activity in the prefrontal‐limbic circuit at rest: Potential imaging markers and treatment predictors in drug‐naive anxiety disorders
Source: CNS Neurosci Ther. 2023 Nov 21;30(4):e14523. doi: 10.1111/cns.14523 (PMC11017453; doi:10.1111/cns.14523)

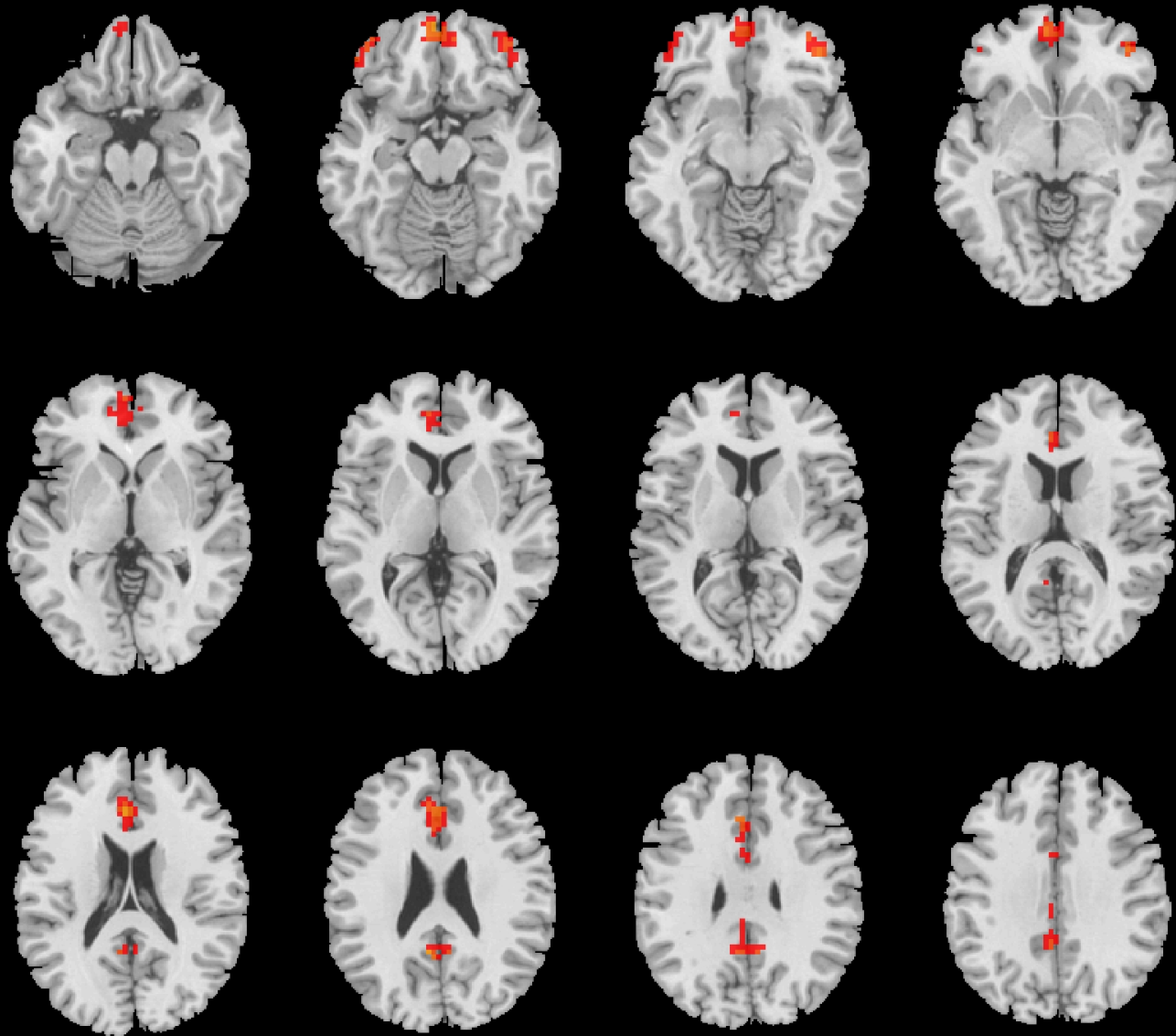

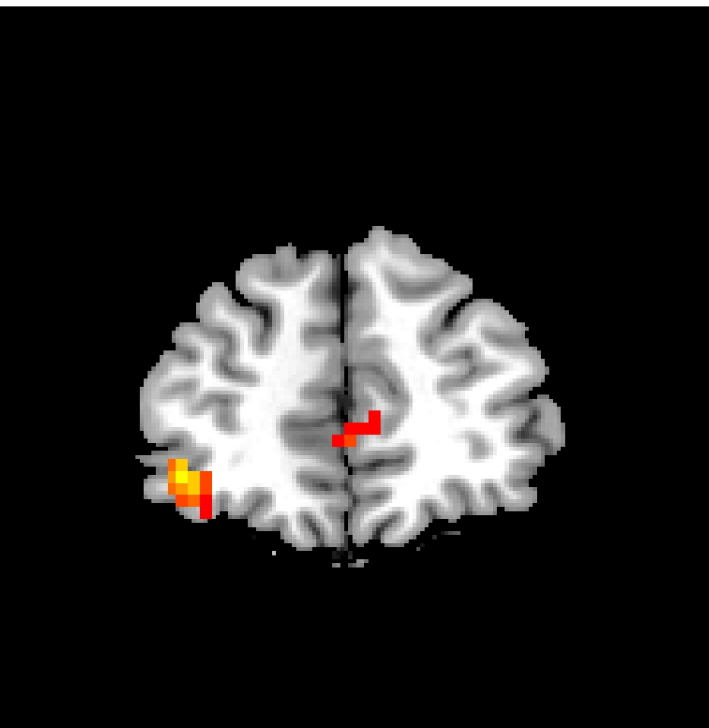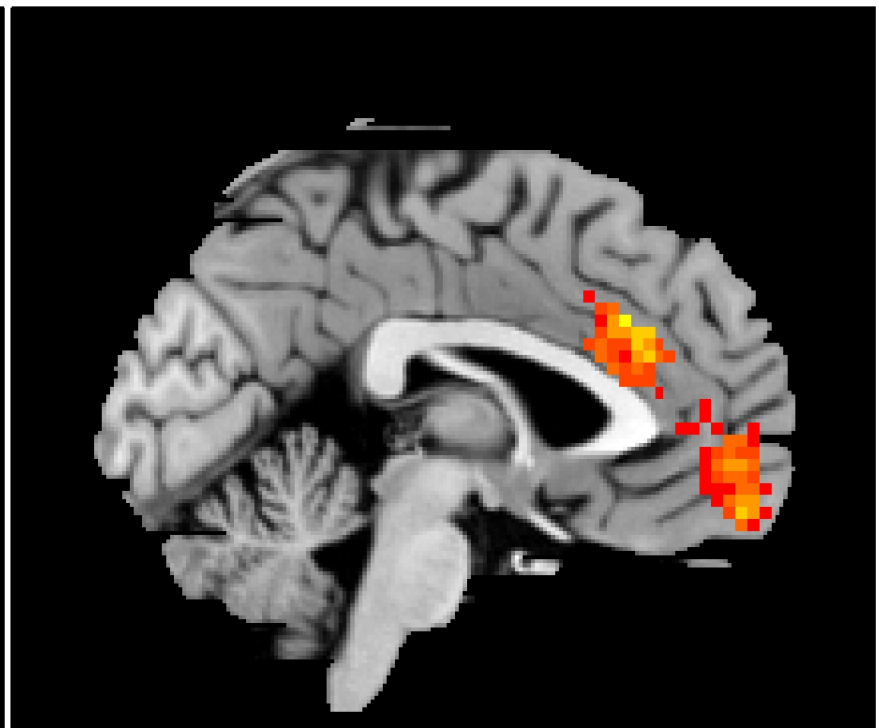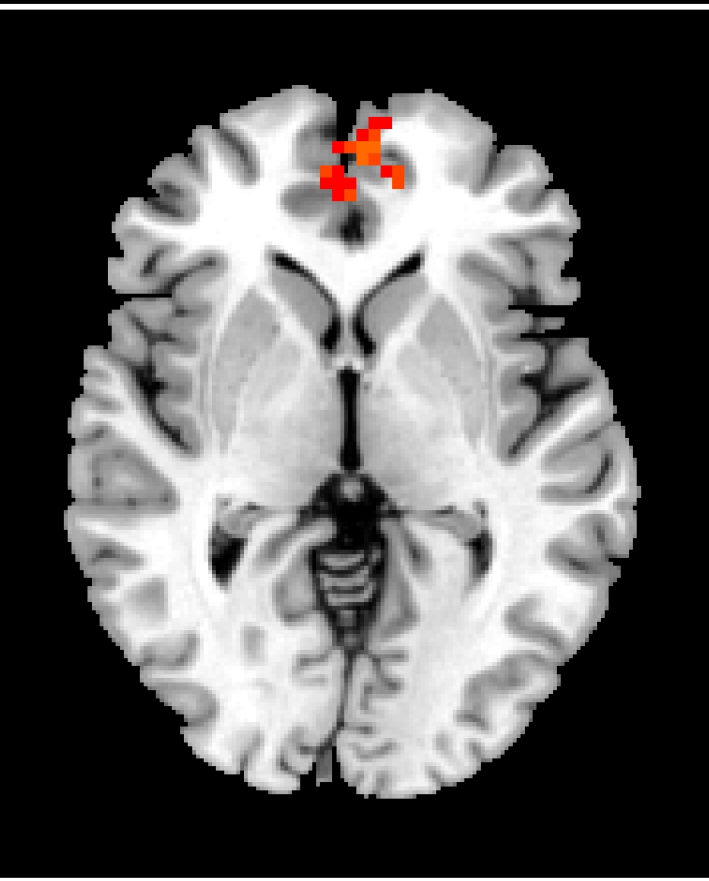

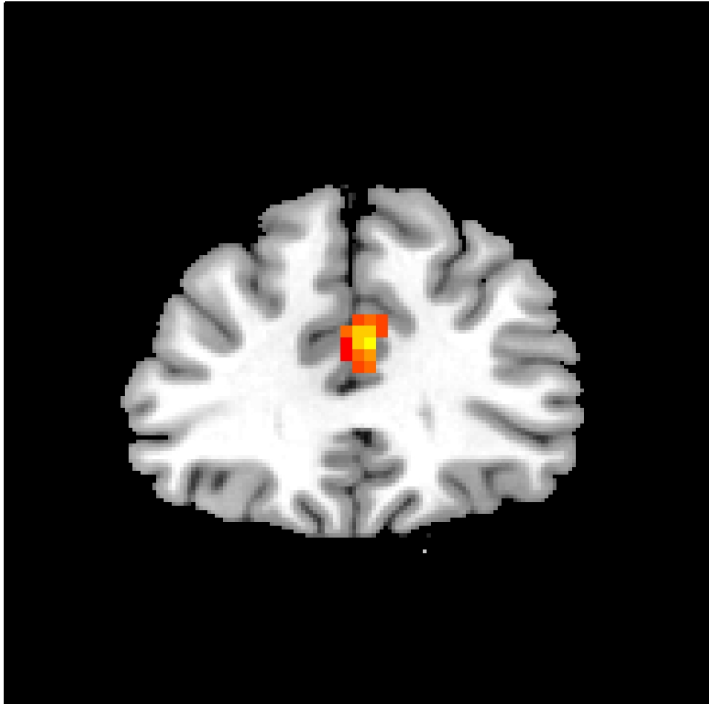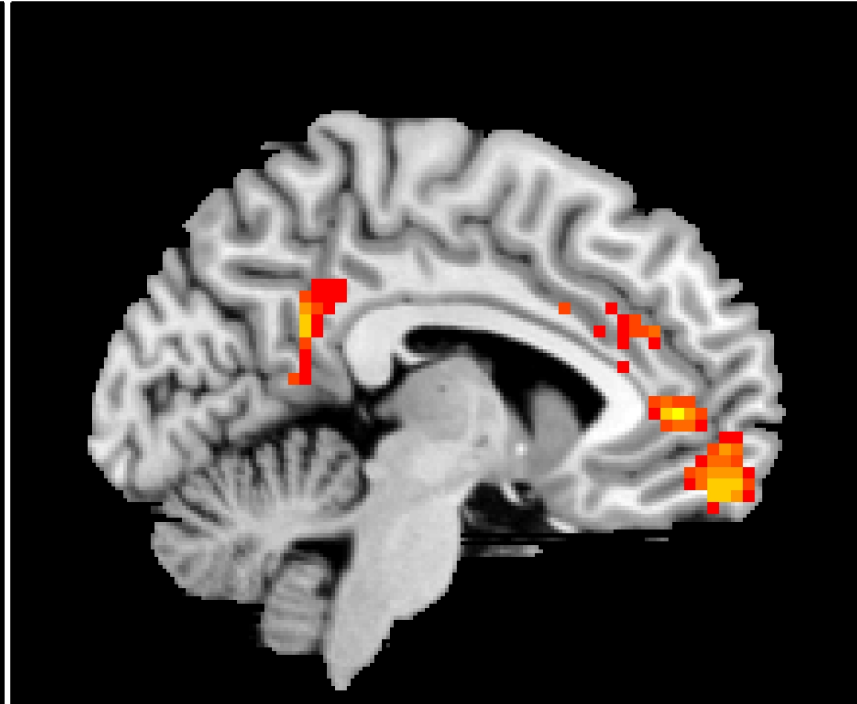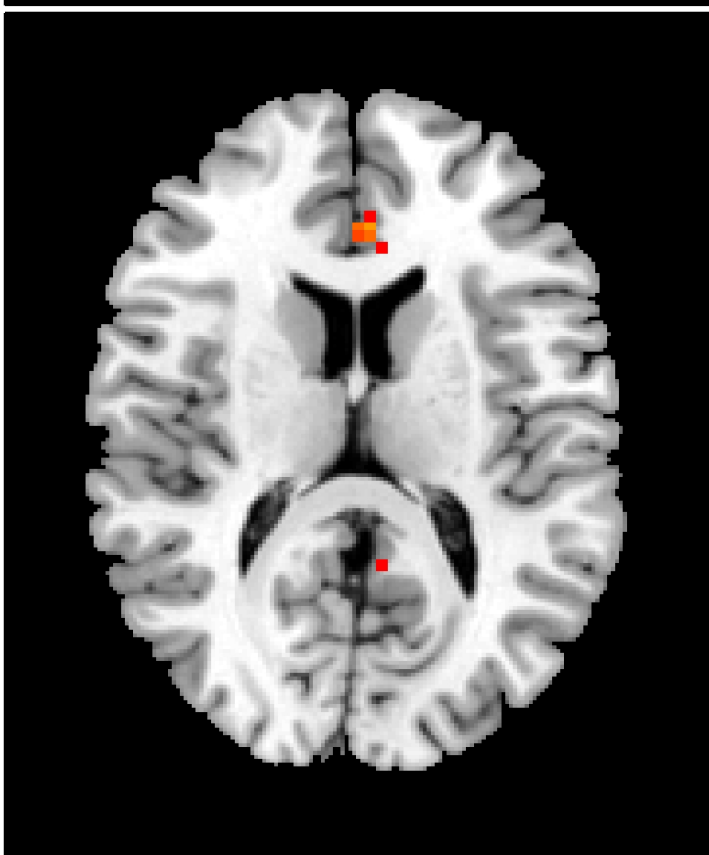

Supplement: Supplementary file 1 — Figure S1. [file CNS-30-e14523-s002.pdf]
