# Supplementary material for: Abnormal regional activity in the prefrontal‐limbic circuit at rest: Potential imaging markers and treatment predictors in drug‐naive anxiety disorders
Source: CNS Neurosci Ther. 2023 Nov 21;30(4):e14523. doi: 10.1111/cns.14523 (PMC11017453; doi:10.1111/cns.14523)

**Supplemental Tables**

Table S1 Brain regions with differences in fALFF between groups

| Cluster location | L/R | Peak (MNI) | | | Number of voxels | T |
| --- | --- | --- | --- | --- | --- | --- |
|  |  | X | Y | Z |  |  |
| **GAD vs HCs** |  |  |  |  |  |  |
| medial OFG /anterior and paracingulate gyrus | L/R | -3 | 57 | 18 | 171 | 5.1941 |
| inferior OFG/ middle OFG | R | 42 | 45 | -9 | 61 | 5.9154 |
| anterior and paracingulate gyrus | L | -3 | 27 | 30 | 70 | 5.6394 |
| **PD vs HCs** |  |  |  |  |  |  |
| medial OFG/ anterior and paracingulate gyrus | L | -3 | 57 | -15 | 131 | 5.9515 |
| anterior and paracingulate gyrus | L | -3 | 33 | 21 | 101 | 5.5775 |
| medial posterior/paracingulate | L | -3 | -54 | 27 | 85 | 5.3722 |
| **GAD vs PD** |  |  |  |  |  |  |
| No |  |  |  |  |  |  |

OFG=orbitofrontal gyrus

Table S2 fALLF changes across patients from baseline to 4 weeks of treatment

| Cluster location | L/R | Peak (MNI) | | | Number of voxels | T |
| --- | --- | --- | --- | --- | --- | --- |
|  |  | X | Y | Z |  |  |
| **GAD group after 4 weeks vs at baseline** |  |  |  |  |  |  |
| Superior OFG /inferior OFG | R | 15 | 15 | -21 | 59 | -4.505 |
| **PD group after 4 weeks vs at baseline** |  |  |  |  |  |  |
| No |  |  |  |  |  |  |

Table S3 ROI results using ALFF index at baseline

|  | | | | | | | |
| --- | --- | --- | --- | --- | --- | --- | --- |
|  | L/R | GAD（n=60） | PD （n=52） | HCs（n=60） | F | Uncorrected P | Post hoc t-tests |
| **Amygdala** |  |  |  |  |  |  |  |
| mAmyg | L | 0.4714±0.5410 | 0.4773±0.4925 | 0.3231±0.3576 | 1.969 | 0.143 |  |
| mAmyg | R | 0.3846±0.4417 | 0.4534±0.4398 | 0.5216±0.5085 | 1.132 | 0.325 |  |
| lAmyg | L | -0.0413±0.2781 | 0.0053±0.3327 | 0.0375±0.3973 | 0.847 | 0.430 |  |
| lAmyg | R | -0.022±0.5108 | -0.0140±0.4668 | 0.1888±0.6950 | 2.340 | 0.099 |  |
| **Hippocampus** |  |  |  |  |  |  |  |
| rHipp | L | 0.2077±0.2816 | 0.2101±0.2859 | 0.2306±0.3604 | 0.058 | 0.943 |  |
| rHipp | R | 0.2098±0.2869 | 0.2940±0.3522 | 0.4642±0.3823 | 7.707 | 0.001 |  |
| cHipp | L | -0.1539±0.2190 | -0.1529±0.1507 | -0.0673±0.3242 | 2.565 | 0.080 |  |
| cHipp | R | -0.2387±0.1682 | -0.1752±0.2966 | -0.0413±0.2174 | 10.337 | 0.000059 | GAD＜HCs， PD＜HCs  P＜0.001，P=0.007 |
| **Insula** |  |  |  |  |  |  |  |
| hyperG | L | -0.2225±0.1699 | -0.2063±0.1808 | -0.0971±0.2788 | 5.272 | 0.006 |  |
| hyperG | R | -0.1877±0.1781 | -0.1344±0.2527 | -0.1133±0.2273 | 1.968 | 0.143 |  |
| vIa | L | -0.2190±0.2815 | -0.1461±0.3158 | -0.0878±0.4145 | 2.012 | 0.137 |  |
| vIa | R | -0.2018±0.2705 | -0.1880±0.2943 | -0.0308±0.3294 | 4.773 | 0.010 |  |
| dIa | L | -0.2370±0.1947 | -0.2167±0.1761 | -0.1362±0.2469 | 3.259 | 0.041 |  |
| dIa | R | -0.2126±0.1991 | -0.2077±0.2341 | -0.072±0.3545 | 5.427 | 0.005 |  |
| vId/vIg | L | 0.1845±0.3026 | 0.1977±0.3604 | 0.3517±0.41883 | 3.445 | 0.034 |  |
| vId/vIg | R | 0.1836±0.3723 | 0.1553±0.2485 | 0.0904±0.3512 | 1.153 | 0.318 |  |
| dIg | L | -0.2146±0.1972 | -0.191±0.2514 | -0.0639±0.2426 | 6.529 | 0.002 |  |
| dIg | R | -0.252±0.223 | -0.2200±0.2218 | -0.1578±0.2147 | 2.719 | 0.069 |  |
| dId | L | -0.2071±0.1907 | -0.1602±0.2269 | -0.0872±0.2427 | 4.087 | 0.019 |  |
| dId | R | -0.2935±0.1686 | -0.2542±0.2099 | -0.1751±0.2294 | 4.862 | 0.009 |  |
| **Cingulate gyrus** |  |  |  |  |  |  |  |
| A23d | L | 0.04181±0.2716 | 0.07900±0.2191 | 0.2689±0.3368 | 11.40 | ＜0.0001 | GAD＞HCs，PD＞HCs  P＜0.001， P＜0.001 |
| A23d | R | 0.3265±0.3392 | 0.3242±0.3284 | 0.4565±0.4321 | 2.215 | 0.112 |  |
| A24rv | L | 0.2640±0.5300 | 0.3011±0.6331 | 0.0246±0.3750 | 5.348 | 0.006 |  |
| A24rv | R | -0.0076±0.2542 | 0.0587±0.3398 | -0.0732±0.3044 | 2.402 | 0.094 |  |
| A32p | L | 0.0762±0.3518 | 0.0667±0.3557 | -0.1058±0.3234 | 5.470 | 0.005 |  |
| A32p | R | 0.1277±0.4065 | 0.1241±0.3194 | 0.2759±0.5151 | 2.051 | 0.132 |  |
| A23v | L | 0.3498±0.3916 | 0.2695±0.3189 | 0.5216±0.4636 | 5.914 | 0.003 |  |
| A23v | R | -0.1469±0.2849 | -0.1552±0.2363 | -0.0077±0.3012 | 4.536 | 0.012 |  |
| A24cd | L | 0.0952±0.2964 | 0.2977±0.4021 | 0.0769±0.3629 | 7.278 | 0.000934 | GAD＜PD，PD＞HCs  P=0.002， P=0.001 |
| A24cd | R | 0.2357±0.4161 | 0.2377±0.3293 | 0.3579±0.3829 | 1.754 | 0.176 |  |
| A23c | L | -0.098±0.2161 | -0.0852±0.2084 | 0.0034±0.2765 | 3.716 | 0.026 |  |
| A23c | R | 0.4626±0.3572 | 0.4561±0.3730 | 0.3186±0.3363 | 2.974 | 0.054 |  |
| A32sg | L | -0.1321±0.3444 | -0.096±0.2934 | 0.1948±0.4386 | 13.507 | ＜0.0001 | GAD＞HCs，PD＞HCs  P＜0.001， P＜0.001 |
| A32sg | R | 0.2909±0.3647 | 0.3396±0.3756 | 0.8713±0.5634 | 29.362 | ＜0.0001 | GAD＞HCs，PD＞HCs  P＜0.001， P＜0.001 |
| **Orbitofrontal lobe** |  |  |  |  |  |  |  |
| A14m | L | 0.2916±0.4262 | 0.2161±0.4387 | -0.0563±0.3442 | 11.741 | ＜0.0001 | GAD＞HCs，PD＞HCs  P＜0.001， P=0.001 |
| A14m | R | -0.0149±0.3126 | -0.0228±0.3738 | 0.0532±0.3634 | 0.847 | 0.431 |  |
| A12/47o | L | -0.0440±0.260 | -0.0845±0.2692 | 0.1078±0.3822 | 5.212 | 0.006 |  |
| A12/47o | R | 0.02438±0.2505 | 0.0637±0.1938 | 0.1261±0.3762 | 5.176 | 0.007 |  |
| A11l | L | 0.0753±0.2039 | 0.0206±0.223 | 0.1276±0.2647 | 2.039 | 0.133 |  |
| A11l | R | 0.1489±0.2095 | 0.0980±0.2565 | 0.2506±0.2515 | 5.143 | 0.007 |  |
| A11m | L | 0.0602±0.4342 | 0.1162±0.5873 | -0.1562±0.5261 | 4.349 | 0.014 |  |
| A11m | R | 0.0488±0.3448 | 0.0496±0.4299 | 0.0036±0.5049 | 0.275 | 0.760 |  |
| A13 | L | -0.256±0.2702 | -0.2170±0.2684 | 0.0783±0.4184 | 18.484 | ＜0.0001 |  |
| A13 | R | -0.2934±0.2795 | -0.2445±0.3412 | 0.1191±0.5163 | 20.215 | ＜0.0001 |  |
| A12/47l | L | -0.3572±0.2534 | -0.3748±0.2799 | -0.1279±0.3669 | 12.453 | ＜0.0001 | GAD＞HCs，PD＞HCs  P＜0.001，P=0.001 |
| A12/47l | R | -0.260±0.2736 | -0.3055±0.2648 | -0.0843±0.4028 | 8.413 | 0.000295 | GAD＞HCs，PD＞HCs  P=0.002，P＜0.001 |

**Supplemental Figures**

**Figure S1**. fALFF differences across groups.

The color bar represents the t values of the group analysis of fALFF.

**Figure S1**
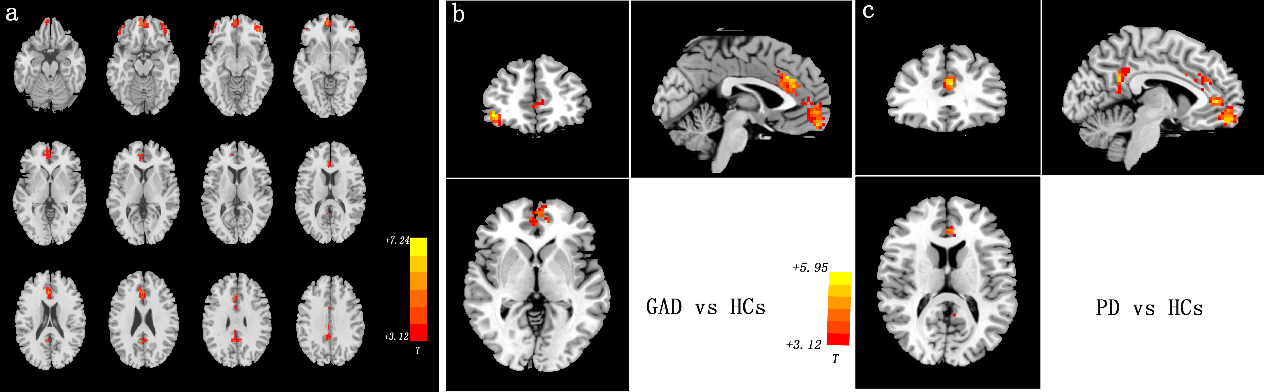

Supplement: Supplementary file 2 — Table S1. Table S2. Table S3. [file CNS-30-e14523-s001.docx]
